# Ochratoxin A Levels in Tissues of Wild Boars (*Sus scrofa*) from Northern Italy

**DOI:** 10.3390/toxins12110706

**Published:** 2020-11-08

**Authors:** Tiziano Iemmi, Alessandro Menozzi, Valentina Meucci, Irene Magnini, Federica Battaglia, Lorella Severino, Andrea Ariano, Simone Bertini

**Affiliations:** 1 Department of Veterinary Science, University of Parma, 43126 Parma, Italy; tiziano.iemmi@unipr.it (T.I.); irene.magnini@studenti.unipr.it (I.M.); simone.bertini@unipr.it (S.B.); 2 Department of Veterinary Science, University of Pisa, 56124 Pisa, Italy; valentina.meucci@unipi.it (V.M.); federica.battaglia@phd.unipi.it (F.B.); 3 Department of Veterinary Medicine and Animal Production, University of Naples Federico II, 80137 Naples, Italy; lorella.severino@unina.it (L.S.); andrea.ariano@unina.it (A.A.)

**Keywords:** ochratoxin A, wild boar, HPLC, liver, muscle, kidney, Wild boar meat from northern Italy is a possible source of OTA

## Abstract

Ochratoxin A (OTA) is a mycotoxin produced by *Aspergillus* and *Penicillium*, capable of contaminating several foodstuffs. OTA damages primarily the kidneys, and is suspected to be a carcinogenic substance, thus maximum levels for OTA in foodstuffs have been established in the EU. Italian Ministry of Health suggested a maximum level of 1 μg/kg OTA in pork meat and derived products. In this study, OTA concentrations in liver, kidney, and muscle of 64 wild boars (*Sus scrofa*) killed in two areas (area A and B) of Parma province (northern Italy), characterized by different habitat types, were assessed by HPLC-FLD technique. OTA was detected in 54% liver, 52% kidney, and 16% muscle samples. OTA levels were significantly higher in liver and kidney compared with muscle, and were above 1 μg/kg in 19 liver, 17 kidney, and 4 muscle samples. OTA levels in wild boars from area A resulted significantly higher with respect to those from area B, suggesting an environmental influence on OTA contamination in wild boars. This study seems to confirm that wild boar meat is a potential source of OTA, thus monitoring the presence of this mycotoxin in game meat might be recommended to prevent risks for human health.

## 1. Introduction

Ochratoxins are a group of mycotoxins produced by fungi of the genera *Aspergillus* and *Penicillium*, almost ubiquitous in the environment. Ochratoxin A (OTA) is the most important and harmful ochratoxin, capable of contaminating several agricultural products and foods, such as barley, sorghum, corn, legumes, coffee beans, dried fruits, spices, beer, wine, cheese, and meat [1]. The presence of OTA in food products of animal origin may be due to the proliferation of fungi and consequent mycotoxin synthesis and release in the alimentary material, but it may also be a consequence of the carry-over phenomenon from contaminated animal feed [2]. After oral intake, OTA is absorbed in the gastrointestinal tract in the non-ionized form and by means of cytochrome P450, forms active metabolites which seem to play a major role in OTA toxicity [2].

OTA is highly bound to albumin in blood, and this, together with the fact that the mycotoxin is subjected to enterohepatic recirculation, contributes to its long elimination half-life, which was found as long as 72–120 h in pigs [3], and up to 35 days in humans [4]. A huge absorption of OTA can cause an acute intoxication with acute renal failure [5], but chronic damage is more common, as a consequence of prolonged oral intake of little amounts. OTA is a nephrotoxic, carcinogenic, teratogenic mycotoxin, and can also be harmful for immune and nervous systems [6].

OTA is responsible of causing endemic nephropathy in pigs [7], which are particularly sensitive to this mycotoxin, but alimentary exposure to OTA seems to cause renal damage in many species, including rodents, dogs, and birds, and even in young ruminants [8].

OTA was also suspected to be involved in the pathogenesis of Balkan endemic nephropathy (BEN) in humans [6,7,9,10], a form of chronic interstitial nephritis which has several similarities with pig nephropathy [8,11]. More recent evidence, however, seems to suggest that OTA alone is unlikely able to induce BEN, but could play a synergistic role together with other toxic substances, such as aristolochic acid and citrinin [12]. Long-term exposure to high levels of OTA has nevertheless been linked to cases of chronic interstitial nephropathy, and also renal neoplasms [8,13,14].

In 1993, the International Agency for Research on Cancer classified OTA in group 2B, “possibly carcinogenic to humans” [15]. Indeed, an exposure to high concentrations of OTA for 48 h was found to induce signs of chromosomal aberrations in human lymphocyte cultures, suggesting that this mycotoxin has a genotoxic potential [16].

Considering the wide geographical diffusion of OTA (mainly northern Europe and Africa) and the high number of potentially contaminated foodstuffs, the international authorities have proposed maximum tolerable intakes for OTA, and the European Union has set maximum accepted levels for OTA in several foodstuffs. A provisional tolerable weekly intake (PTWI) of 120 ng/kg bw for OTA has been established by the European Food Safety Authority, based on its nephrotoxic properties in pigs, the most sensitive animal species [17]. PTWI was established taking into consideration the LOAEL (lowest observed adverse effect level) value of 8 μg/kg bw/day for early markers of renal toxicity. Health Canada, on the other hand, established a markedly lower intake limit, a tolerable daily intake (TDI) of 3 ng/kg bw, based upon the NOAEL (no observed adverse effect level) in place of the LOAEL, considering OTA as a non-threshold carcinogen, in addition to the fact that OTA half-life is longer in humans than in pigs [18,19].

Until now, a maximum level of OTA in meats or other foods of animal origin has however not been set at the European level. OTA concentrations ranging from 0.1 to 103.7 μg/kg have been detected in food products such as pork meat, raw ham, sausages, and salami [20,21]. Indeed, non-ruminant species, such as the pig, tend to accumulate OTA due to the long half-life of the toxin, thus foodstuff of swine origin is a potentially relevant source of OTA, representing a possible risk for the consumer’s health. In addition, OTA is a very stable molecule, therefore normal food processing techniques are not able to significantly reduce its concentration [22].

In Italy, the Ministry of Health has recommended a precautionary maximum level of 1 μg/kg of OTA in pork meat and derived products [23].

This study was carried out in order to evaluate the concentrations of OTA present in muscle, liver, and kidneys of wild boars (*Sus scrofa*), since the meat of this animal is frequently consumed throughout Italy. Wild boars represent one of the most hunted game species in Italy, both for the engaging type of hunting, and for the delicacy of their meats; for example, in the 2017–2018 season, 28,013 animals were hunted in the Emilia Romagna Region (the geographical area where the study was conducted), and an estimated amount of meat ranging from 750,000 to 650,000 kg was destined to human consumption. For such reasons, monitoring OTA levels in wild boar meat could be of relevance in order to protect consumers’ health. Two similar studies have been previously conducted in southern [24] and central [25] Italy, thus we decided to extend the investigation about OTA levels in wild boars to the north of the country, since environmental variability might influence the level of contamination by this mycotoxin.

## 2. Results 

The analytical method has shown to be suitable for accurate quantitative determination of OTA in different tissues of wild boars. Results of the validation study are reported in Table 1.

Recoveries of the analytical method were higher than 86% for all the matrices. Intra- and inter-day repeatability expressed as relative standard deviation was less than 15%. The limit of detection (LOD) and limit of quantification (LOQ) were 0.01 and 0.05 μg/kg for all tissues. The method has shown to be suitable for accurate quantitative determination of OTA in different tissues of wild boars.

Overall, of the 64 wild boars sampled, 41 were females (9 of young and 32 of adult age-class), while 23 were males (13 of young and 10 of adult age-class).

Measurable concentrations of OTA in liver, kidney, and muscle were detected in 54% (*n* = 34), 52% (*n* = 33), and 16% (*n* = 10) of the wild boars, respectively. Overall median OTA levels in wild boars of both areas (area A + area B) in liver and kidney samples were, respectively, 0.22 μg/kg (<LOD–7.80) and 0.28 μg/kg (<LOD–7.70); both these concentrations resulted significantly higher (*p* < 0.001) compared with median OTA level detected in muscle, which resulted <LOD μg/kg (<LOD–1.88) (Table 2, Table 3 and Table 4).

No significant differences in OTA levels in the various tissues were found comparing wild boars of different sex or age-class (Table 2 and Table 3). 

A statistically significant positive correlation was observed between the levels of OTA detected in kidneys and those measured in liver (r = 0.552, *p* < 0.001) and muscle (r = 0.30, *p* = 0.013). Moreover, OTA levels in liver samples were also positively correlated to those detected in muscle (r = 0.366, *p* = 0.003).

A moderate negative correlation between wild boar weight and hepatic OTA levels was discovered (r = − 0.26; *p* = 0.038), whereas no significant correlation between OTA levels in muscle or kidney tissue and subject weight was found. 

**Figure 1:**
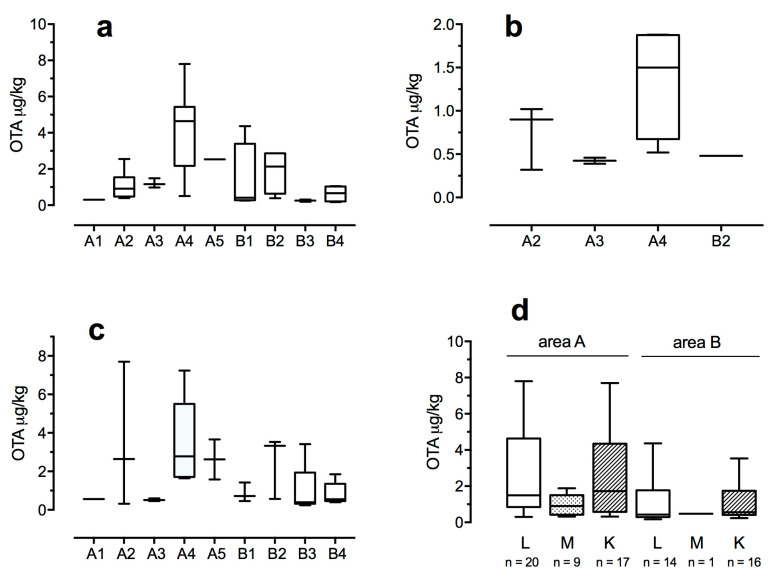
OTA levels in liver (**a**), muscle (**b**), and kidney (**c**) samples with detectable concentrations of OTA (>LOD) in wild boars from different sub-areas (A1–A5; B1–B4). OTA levels in liver (L), muscle (M), and kidney (K) samples with detectable concentrations of OTA (>LOD) in wild boars from area A and B (**d**); n indicates the number of samples with OTA > LOD.

In the 34 liver samples with detectable levels of OTA, the mycotoxin levels ranged from 0.17 to 7.80 μg/kg, exceeding the recommended maximum level of 1 μg/kg in 19 subjects (Figure 1a,d). In the 33 kidney samples with measurable OTA levels, the concentration range was 0.24–7.70 μg/kg, with 17 wild boars in which OTA concentration was above 1 μg/kg (Figure 1c,d). Conversely, only 10 muscle samples showed OTA levels above LOD, ranging from 0.32 to 1.88 μg/kg, with only 4 wild boars with an OTA level above 1 μg/kg (Figure 1b,d). 

Samples from wild boars killed in area A showed significantly higher OTA levels in liver, kidneys, and muscle, compared with those measured in animals from area B (Table 4). Wild boars killed in sub-area A4 showed significantly higher OTA levels in liver and kidneys compared with the other sub-areas of area A, and to overall OTA levels: 4.63 μg/kg (*p* = 0.002 and *p* = <0.001) and 2.13 μg/kg (*p* = 0.042 and *p* = 0.001), respectively (Table 4). Furthermore, OTA concentrations measured in muscle samples of wild boars from A4 sub-area were significantly higher (*p* = 0.006) with respect to overall OTA levels in this tissue (Table 4).

## 3. Discussion

In the hunting activity, wild boars are sought-after preys, primarily for the delicacy of their meat, and although muscle is the most consumed part, either fresh or after the production of cured meats, the liver is also much appreciated and frequently used in local cooking recipes. The production of cured meats requires a dehydration process of the meat, with a loss in weight of 30–40%, resulting in an increase in OTA concentration. This may therefore lead to an enhanced health risk associated with the consumption of these products [26], even if the starting levels of OTA in fresh meat are low. Moreover, the addition of flavoring substances, such as spices, which are also subjected to contamination by OTA, may represent an additional source of mycotoxin [27].

In the present study, OTA was detected in 54% of liver samples and 52% of kidney samples, whereas a lower percentage of muscle samples (16%) showed OTA levels above LOD.

While OTA levels in kidney and liver seem to be positively correlated to those found in muscle samples, no correlation between OTA levels in muscle or kidney tissue and subject weight was found. This result could be due to the dilution effect on OTA concentration due to subject’s body mass; in addition, wild boars with a higher body mass could have greater degradation ability [28], and access to less contaminated food sources in relation to social behavior and foraging hierarchy [29]. However, this finding is in accordance with previous studies, since body weight was found to be negatively correlated to OTA levels in kidneys [24,30]. In the present study, OTA levels in different tissues appear also being not influenced by sex or age-class.

In previous studies conducted on wild boars from the central part of Italy [25], or in the south of Italy [24], lower maximum levels of OTA were found, with respect to our study in liver, kidney, and muscle samples (1.93 and 2.00 μg/kg vs. 7.80 μg/kg, 3.23 and 3.90 μg/kg vs. 7.70 μg/kg, 0.77 and 1.30 μg kg vs. 1.88 μg/kg, respectively). 

Among the wild boars enrolled in this study, 4 muscle samples (6.3%) and 19 liver samples (29.7%) out of 64 showed OTA levels above the recommended maximum concentration of 1 μg/kg; conversely, in previous studies, a lower degree of contamination by OTA in muscle and liver samples was detected. In fact, in the study by Bozzo et al. [24], respectively 1 muscle (4.3%) and 3 liver (13.0%) samples out of 23 showed an OTA level above 1 μg/kg, while in the study by Luci et al. [25] OTA was below this threshold concentration in all 48 muscle samples, and only 6 liver samples (12.5%) had OTA levels above the limit. Moreover, the maximum OTA concentration found in our study in muscle and liver samples (1.88 and 7.80 μg/kg, respectively) was higher compared with previous studies by Bozzo et al. (1.30 and 2 μg/kg) [24] and Luci et al. (0.77 and 1.54 μg/kg) [25].

Even though these concentrations of OTA may not seem to represent a risk for the consumer’s health, also considering that wild boar meat is not eaten as frequently as other types of meat, the results of this study confirm that wild boars are a possible source of food with OTA concentrations above the recommended safe level. Moreover, a great part of wild boar meat that comes from hunting activity is consumed by a restricted group of people represented by hunters and their relatives, thus they are more exposed to contaminants present in game meat compared with average population. However, assessing the amount of wild boar meat consumed by hunters in comparison to general population would be crucial to evaluate the level of risk for consumer’s health related to OTA contamination, and the lack of this evaluation may represent a limitation of the study.

Interestingly, the results of the present study may suggest that the contamination levels by OTA in wild boars might be influenced by the geographical area. Samples from wild boars killed in area A showed significantly higher OTA levels in liver, kidneys, and muscle, compared with those measured in animals from area B. It is noteworthy that in one of the five sub-areas of area A (A4), there were significantly higher OTA levels in liver and kidneys compared with the other sub-areas of the area A, and compared with overall OTA levels. Furthermore, OTA concentrations measured in muscle samples of wild boars from A4 sub-area were significantly higher (*p* = 0.006) compared with overall OTA levels in this tissue. In addition, three of the four wild boars that had OTA levels above 1 μg/kg in muscle samples were killed precisely in this sub-area. 

The habitat of area A is mainly constituted by riparian bush, whereas area B is characterized by woods of deciduous Fagaceae trees. Wild boars are omnivorous animals, and feed primarily on foods of vegetable origin with a high starch content, such as the underground parts of plants (roots, tubers, bulbs), fruits and seeds of arboreal and herbaceous plants, mushrooms, but eat also eggs, carcasses, invertebrates, and other small preys [31]. This poor level of food specialization allows them to colonize a wide range of habitats, by exploiting different food resources [32]. The environmental differences between area A and area B could have influenced the availability of food sources with different levels of OTA contamination, both in qualitative and quantitative terms, and this might explain the significant difference in OTA levels detected in wild boars from different areas, even though the two areas are indeed not far from each other.

## 4. Conclusions

The present study reinforces the hypothesis that wild boar meat is a possible source of OTA for humans. Moreover, the different availability of food sources due to environmental variability may be able to influence the presence of OTA in the diet of wild boars. Since there is no control on the food consumed by wild animals, and the toxic effects caused by chronic exposure to this mycotoxin have not been fully elucidated, a possible risk for human consumers cannot be ruled out, bolstering the need to establish a more thorough legislation framework for contaminants in game meat.

## 5. Materials and Methods

### 5.1. Samples

A total of 64 wild boars were killed by park rangers during the provincial culling plan from November 2018 to February 2019 in two areas of the province of Parma, Emilia Romagna, northern Italy. The culling took place during the normal boar hunting season, which usually begins in September and ends in March.

**Figure 2:**
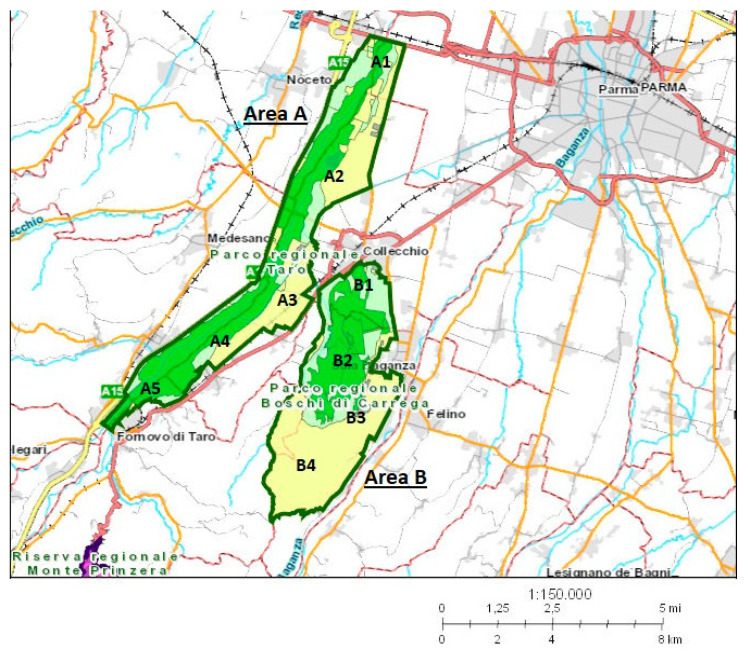
Cartographic image of the two geographic areas (area A and area B) of wild boar sampling, in the province of Parma, northern Italy. In both, area A and area B sub-areas indicating the sampling zones are reported (A1–A5; B1–B4).

The territories occupied by the “Taro Regional Park” and by the “Boschi di Carrega Regional Park” were respectively named area A (20 km^2^) and area B, (12.7 km^2^). The two areas are geographically neighboring, but differ from one another for environmental conditions, and are mutually isolated by the presence of a highway, an infrastructure that represents a hardly surmountable border for ungulates (Figure 2). Five sub-areas (A1–A5), indicating the specific capture sites, are identifiable in area A, and four in area B (B1–B4); 26 wild boars were sampled in area A and 38 in area B. 

The carcasses were immediately brought to a slaughterhouse, where the evisceration was performed, in accordance with the EU regulations [33]. For each animal, a data sheet reporting the day and area of abatement, weight, sex, and age-class (young or adult) was compiled. Age-class was assigned based on the pigmentation of the coat of the animal: light brown-reddish coat for young (0–12 months), and dark brown coat for adult subjects (>12 months). Samples of liver, muscle (biceps femoris), and kidney were collected from each wild boar, individually packed and stored at −20 °C until the laboratory investigations were carried out. The laboratory analyses for OTA level assessment were performed at the Pharmacology and Toxicology laboratory of the Department of Veterinary Sciences of the University of Pisa.

### 5.2. Reagents

OTA (10 μg/mL in acetonitrile) and ochratoxin B (OTB) (10 μg/mL in acetonitrile) reference standard were purchased from Sigma (Milan, Italy). Working solutions were prepared by diluting the stock solution with the mobile phase consisting of a methanol/sodium phosphate buffer (pH 7.5) 60/40% *v*/*v*. HPLC-grade water, methanol, ethyl acetate, and acetonitrile were purchased from VWR (Milan, Italy).

### 5.3. Chromatographic Method

OTA concentrations have been measured by using the HPLC method of Monaci et al. [34], partially modified. The chromatographic system consisted of a Jasco 880 pump and a Jasco 821 fluorescence detector (Jasco, Tokyo, Japan). JascoBorwin software was used for data processing. The excitation wavelength (λ_ex_) and emission wavelength (λ_em_) were set at 380 and 420 nm, respectively. The reversed-phase column was a HAISIL HL, C_18_, 5 μm, 150 mm × 4.6 mm (Higgins Analytical, USA). The column was kept at room temperature. The HPLC was operated with a mobile phase system consisting of a methanol/phosphate buffer solution pH 7.5 (0.03 M Na_2_HPO_4_, 0.007 M NaH_2_PO_4_) 60/40% *v*/*v* at flow rate of 1 mL/min.

### 5.4. Samples Preparation

Samples of muscle (5 g), liver (5 g or less if not available), and kidney (1 g) were obtained from each wild boar tissue sample, and homogenized with 5 mL of phosphoric acid 1 M using an Ultra Turrax T25 homogenizer for a few minutes. Internal standard OTB (100 μL, 100 ng/mL) was added. The homogenate was transferred into a centrifuge tube, extracted with 5 mL of ethylacetate, vortexed for 1 min, shaken for 10 min on horizontal shaker, and then centrifuged for 10 min at 1258× *g*. The organic phase was removed, the residue re-extracted, as above, and the organic phases combined. The volume of the organic phase was reduced to approximately 5 mL and back-extracted with 5 mL of NaHCO_3_ pH 8.4, vortexed for 1 min, and centrifuged for 10 min at 1258× *g*. The aqueous extract was acidified to pH 2.5 with H_3_PO_4_ 85% and briefly sonicated to strip the CO_2_ formed. OTA was finally back-extracted into 5 mL ethylacetate, vortexed for 1 min, and centrifuged for 10 min at 1258× *g*; the organic phase was evaporated to dryness under nitrogen stream, reconstituted in 500 μL of mobile phase, and a 100 μL aliquot injected.

### 5.5. Spiked Samples

Samples spiked before extraction were used to check the performance of the extraction and clean-up procedure and to obtain validation parameters. Spiking solutions of OTA and OTB were prepared daily by dilution with HPLC mobile phase. For samples of muscle, kidney, and liver, after thoroughly mixing for 30 min, the OTA and OTB fortified homogenate was left for at least 2 h at room temperature to enable equilibration and used to assay the cleaning procedures prior to HPLC analysis. 

### 5.6. Method Validation

The HPLC-FLD method was validated according to EU criteria for the confirmatory methods for contaminants [24] by evaluating the following: specificity, recovery, linearity, LOD and LOQ, repeatability and reproducibility. A limit of 1 μg/kg (1 ppb) OTA in pork meat and derived products was established by the Italian Ministry of Health in 1999 [35]. The validation procedure was performed taking into account the value of 1 μg/kg OTA.

**Figure 3:**
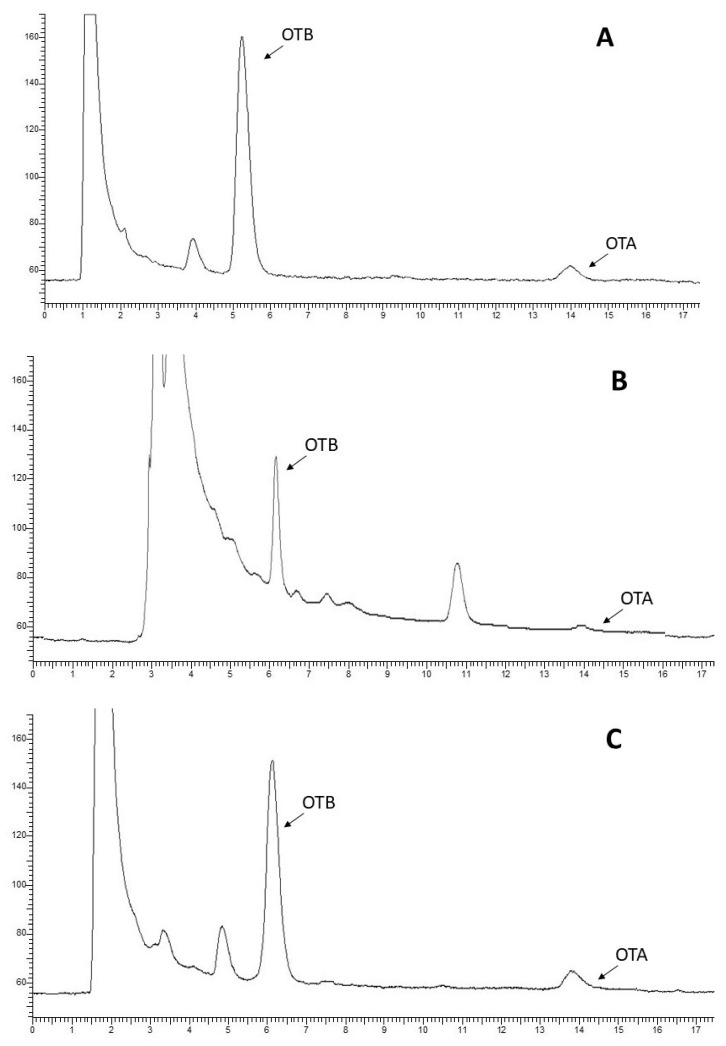
HPLC-FLD chromatograms of liver (**A**), muscle (**B**), and kidney (**C**) naturally contaminated with OTA.

The linearity was evaluated by spiking muscle, liver, and kidney samples with OTA at 0.05, 0.1, 0.5, 1, 2.5, and 5 μg/kg and analyzing them using the extraction and HPLC-FLD method. The experiment was repeated three times. The repeatability was tested by analyzing muscle, liver, and kidney samples spiked with OTA at the levels of 0.1 μg/kg, 1 μg/kg, and 5 μg/kg. All samples were measured in triplicate on the same day. For the within-laboratory reproducibility test, each of the contamination levels was tested in triplicate over a period of five days. The results of these experiments were also used for the determination of the recovery. The LOD and LOQ were determined by the signal-to-noise approach, defined at levels resulting in signal-to-noise ratios of 3 and 10, respectively. The analytical response and the chromatographic noise were measured from the chromatogram of a blank sample extract (1 mL) to which an OTA solution was added. Chromatograms of muscle, liver, and kidney samples of one wild boar naturally contaminated by OTA are shown in Figure 3.

### 5.7. Statistical Analysis

Statistical analysis was performed with GraphPad Prism version 7 software (GraphPad Software Inc., La Jolla, CA, USA). All data were tested for normality by means of the Kolmogorov–Smirnov test. Since data did not prove to be normally distributed, the results were expressed as median and range. Mann–Whitney *U* Test was employed to evaluate the significance of the difference among groups of data, according to different tissues, geographical areas, sex, and age-class. Linear regression analysis and Spearman correlation coefficient analysis were used to assess the correlation between OTA concentrations in muscle, liver, and kidney and weight of wild boars. The ranges of correlation power were r ≥ 0.8; 0.6 ≤ r < 0.8; 0.3 ≤ r ≤ 0.5, and r ≤ 0.2 for strong, moderately strong, moderate, and weak correlation, respectively. A value of *p* < 0.05 was considered statistically significant.

## Figures and Tables

**Table 1:** Validation parameters of HPLC method according to [24]; (LOD = limit of detection, LOQ = limit of quantification, r^2^ = coefficient of correlation, SD = standard deviation, RSD = relative standard deviation).

Parameters		Muscle	Liver	Kidney
LOD (μg/kg)		0.01	0.01	0.01
LOQ (μg/kg)		0.05	0.05	0.05
r^2^		0.998	0.999	0.995
Repeatability				
0.1 μg/kg (*n* = 3)	Mean concentration ± SD RSD (%)	0.092 ± 0.006 6.20	0.088 ± 0.003 3.64	0.086 ± 0.005 6.15
1.0 μg/kg (*n* = 3)	Mean concentration ± SD RSD (%)	0.83 ±0.04 4.82	0.92 ± 0.11 11.80	0.88 ± 0.08 9.10
5.0 μg/kg (*n* = 3)	Mean concentration ± SD RSD (%)	5.03 ± 0.15 3.03	4.86± 0.35 7.22	4.73 ± 0.11 2.33
Reproducibility				
0.1 μg/kg (*n* = 15)	Mean concentration ± SD RSD (%)	0.091 ± 0.007 8.01	0.088 ± 0.008 8.65	0.086 ± 0.005 6.17
1.0 μg/kg (*n* = 15)	Mean concentration ± SD RSD (%)	0.92 ± 0.12 13.3	0.96 ± 0.13 14.03	0.84± 0.08 9.50
5.0 μg/kg (*n* = 15)	Mean concentration ± SD RSD (%)	4.87 ± 0.17 3.45	4.76 ± 0.21 4.48	4.75 ± 0.11 2.42
OTA Recovery %				
0.1 μg/kg (*n* = 15)		90.78 ± 7.27	88.44 ± 7.65	86.89 ± 5.30
1.0 μg/kg (*n* = 15)		91.67 ± 12.3	96.33 ± 13.5	86.44 ± 8.00
5.0 μg/kg (*n* = 15)		97.40 ± 3.36	95.18 ± 1.42	95.02 ± 2.31
				
OTB Recovery %				
20.0 μg/kg (*n* = 15)		95.30 ± 4.56	91.00 ± 3.21	91.24 ± 5.10

**Table 2:** Ochratoxin A (OTA) concentrations (μg/kg) in liver, muscle, and kidneys of wild boars according to sex. All data are expressed as median and range. LOD: limit of detection.

Sex	*n*	Liver	Muscle	Kidney
Males	23	0.50 (<LOD–5.75)	<LOD (<LOD–1.88)	0.45 (<LOD–7.70)
Females	41	<LOD (<LOD–7.80)	<LOD (<LOD–1.14)	<LOD (<LOD–5.66)
Total	64	0.22 (<LOD–7.80) *	<LOD (<LOD–1.88)	0.28 (<LOD–7.70) *

* p < 0.001 vs. Muscle.

**Table 3:** Median and range of OTA concentrations (μg/kg) in liver, muscle, and kidneys of wild boars according to age-class. All data are expressed as median and range. LOD: limit of detection.

Age Class	*n*	Liver	Muscle	Kidney
Young	22	0.34 (<LOD–5.75)	<LOD (<LOD–1.86)	0.39 (<LOD–3.66)
Adult	42	0.09 (<LOD–7.80)	<LOD (<LOD–1.88)	0.12 (<LOD–7.70)
Total	64	0.22 (<LOD–7.80) *	<LOD (<LOD–1.88)	0.28 (<LOD–7.70) *

* p < 0.001 vs. Muscle.

**Table 4:** OTA concentrations (μg/kg) in liver, muscle, and kidneys of wild boars according to different sampling areas (area A and area B) and sub-areas (A1–A5; B1–B4). All data are expressed as median and range. LOD: limit of detection.

Sampling Area	Liver	Muscle	Kidney
A1 (*n* = 2)	0.15 (<LOD–0.30)	<LOD (<LOD–<LOD)	0.28 (<LOD–0.56)
A2 (*n* = 7)	0.80 (<LOD–2.55)	<LOD (<LOD–1.02)	<LOD (<LOD–7.70)
A3 (*n* = 4)	1.07 (<LOD–1.49)	0.20 (<LOD–0.46)	0.49 (<LOD–0.60)
A4 (*n* = 10)	4.63 (<LOD–7.80) ^a,b^	<LOD (<LOD–1.88)	2.13 (<LOD–7.24) ^c,d^
A5 (*n* = 3)	<LOD (<LOD–2.53)	<LOD (<LOD–<LOD)	1.58 (<LOD–3.66)
Area A (*n* = 26)	1.09 (<LOD–7.80) *^,^^§^	<LOD (<LOD–1.88) ^§^	0.58 (<LOD–7.70) *^,^^§^
B1 (*n* = 10)	<LOD (<LOD–4.37)	<LOD (<LOD–<LOD)	<LOD (<LOD–1.42)
B2 (*n* = 5)	1.41 (<LOD–2.87)	<LOD (<LOD–0.48)	0.57 (<LOD–3.53)
B3 (*n* = 10)	<LOD (<LOD–0.31)	<LOD (<LOD–<LOD)	0.12 (<LOD–3.42)
B4 (*n* = 13)	<LOD (<LOD–1.05)	<LOD (<LOD–<LOD)	<LOD (<LOD–1.85)
Area B (*n* = 38)	<LOD (<LOD–4.37)	<LOD (<LOD–0.48)	<LOD (<LOD–3.53)
Total (area A + B) (*n* = 64)	0.22 (<LOD–7.80) *	<LOD (<LOD–1.88)	0.28 (<LOD–7.70) *

* p < 0.001 vs. Muscle; ^§^ *p* < 0.001 vs. Area B; ^a^ *p* = 0.002, and ^c^ *p* = 0.042 vs. other sub-areas; ^b^ *p* < 0.001, and ^d^ *p* = 0.001 vs. total OTA level.
